# Great Saphenous Vein Lumen: Intimal Openings

**DOI:** 10.21470/1678-9741-2024-0382

**Published:** 2025-12-10

**Authors:** Andrzej Loesch

**Affiliations:** 1 Centre for Rheumatology and Connective Tissue Diseases, Research Department of Inflammation and Rare Diseases, Division of Medicine, University College London, London, United Kingdom

**Keywords:** Saphenous Vein, Vasa Vasorum, Coronary Artery Bypass

## Abstract

This review discusses the morphological characteristics of the human great saphenous vein (SV) harvested for coronary artery bypass grafting (CABG). It focuses on the vein’s luminal intima, which was examined using laser confocal microscopy (LCM), transmission electron microscopy (TEM), and scanning electron microscopy (SEM). Summarised findings are: (1) LCM observations revealed that the vessel-like profiles, formed by the intima of the peripheral parts of SV luminal folds, may create a false impression that these are vasa vasorum vessels terminating at the vein lumen. (2) The SV luminal intima displays openings ranging from about 5 µm to 20 µm. Among these, larger openings (> 10 µm) are recognized as openings of small tributary branches rather than vasa vasorum vessels donating to SV lumen. It is suggested that these vessel openings are involved in the retrograde blood flow into the SV graft wall after CABG. In contrast, openings < 10 µm, or even those < 5 µm, did not show obvious vascular characteristics, suggesting these structures might have another physiological function. (3) In addition to the abovementioned openings, narrow, elongated intimal openings approximately 3 µm by 30 µm in size can be seen at the SEM level; these likely represent the entrances to the small folds detected by TEM in the inner media of the SV. Communication between the SV lumen and the vein vasa vasorum seems crucial for the anti-ischaemic protection of the vein as coronary graft. This issue, including the role of intimal openings, may require further investigation.

## INTRODUCTION

**Table:** 

Abbreviations, Acronyms & Symbols				
A	= Adventitia		LM	= Light microscopy
CABG	= Coronary artery bypass grafting		Lu	= Lumen
CON	= Conventional		Me	= External layer of the media
Ct	= Connective tissue		Mi	= Internal layer of the media
En	= Endothelium		NT	= No-touch
fo	= Small branching fold		SEM	= Scanning electron microscopy
Fo	= Inward luminal fold		sm	= Smooth muscle cell
In	= Intima		SV	= Great saphenous vein
iNOS	= Inducible nitric oxide synthase		TEM	= Transmission electron microscopy
LCM	= Laser confocal microscopy		va	= Vasa vasorum

**Fig. 1:**
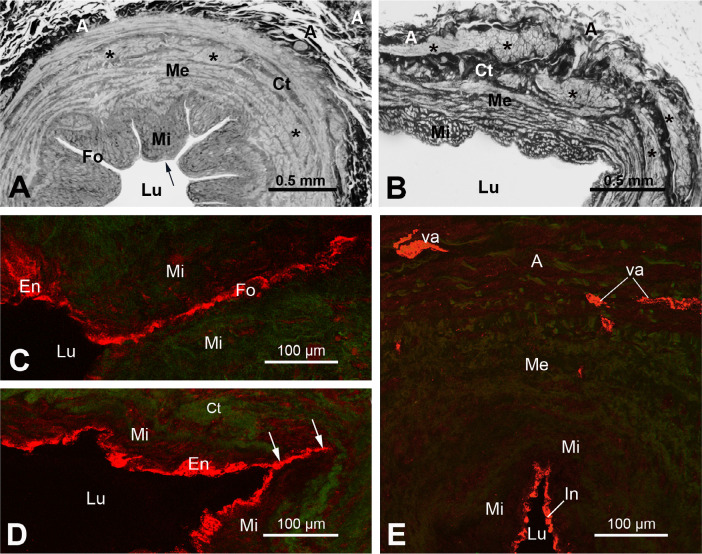
Light microscopy (LM) and laser confocal microscopy (LCM) of transverse sections of proximal segment of human great saphenous vein (SV) harvested by the no-touch (NT) or conventional (CON) method. A) The LM Araldite semithin sections (~ 2 µm) of NT-SV (originally stained with toluidine-blue) show a lumen (Lu) and the internal layer of the media (Mi) which is covered by the intima (arrow); between Mi are seen inward luminal folds (Fo). The external layer of the media (Me) contains bundles of smooth muscle (*) and connective tissue (Ct); adventitia (A) is at the abluminal site of the vein wall. B) The LM section of distended CON-SV shows distended luminal folds. C) The LCM section of NT-SV immunolabelled for inducible nitric oxide synthase (iNOS) shows iNOS-positive (red) endothelium (En) of Fo giving a false impression of a blood vessel being there. D) LCM section of CON-SV shows iNOS-positive En in the partially distended luminal fold; a non-distended part of the fold might resemble a blood vessel (two arrows). E) LCM section of NT-SV shows no connectivity between iNOS-positive vasa vasorum (va) and the iNOS-positive luminal intima (In). Note that for C), D), and E), the main steps of immunolabelling were carried out on 30 µm frozen transverse-sections and involved: (1) fixation with 4% paraformaldehyde; (2) incubation with a rabbit polyclonal antibody to iNOS (Santa Cruz Biotech); (3) incubation with a goat antirabbit immunoglobulin G Alexa Fluor® 568 (Molecular Probes); (4) embedment in Citifluor; and (5) examination at a LCM: Leica DMRBE with SPZ confocal head. The images were collected at 1.5 µm intervals and then merged as maximal projection. It is acknowledged that A) and B) images are modified from Ahmed et al.[4],2004; C), D), and E) are from A. Loesch unpublished study.

When the first coronary artery bypass grafting (CABG) surgeries were performed with the great saphenous vein (SV) as a graft, the focus was on treating coronary artery disease^[1]^. Therefore, it is not surprising that little consideration, if any, was given to the possible role of the vein periand para-vascular components. This seems to be the case, as stripping of the vein from its pedicle and distending the vein (the conventional [CON] method of harvesting) undoubtedly caused damage or distortion to the vein structure, including the intima, media, and adventitia, as well as its vasa vasorum system^[2-5]^. The introduction of the “no-touch” (NT) method of SV harvesting^[2,3]^ enabled better structural and functional preservation of SV as coronary graft. This NT method of harvesting of SV also resulted in other beneficial properties, namely preventing the graft from kinking and twisting when it is excessively lengthy or even protecting it from injury caused by vascular clamping^[2,3]^. Here, Figures 1A and B demonstrate general morphological differences between NT-SV and CON-SV graft preparations as can be seen at the light microscopy (LM) level.

The general morphology and/or substructure of SV have been extensively studied using a variety of techniques, including LM, laser confocal microscopy (LCM), transmission electron microscopy (TEM), scanning electron microscopy (SEM), and, in some cases, also employing immunohistochemical methods. One of the areas of research, which is related to the subject of this review article, concerns the question of the vasa vasorum in relation to the luminal compartment of SV. Here must be mentioned the elegant SEM studies revealing the structural details and complexity of the vasa vasorum of the human SV^[6-11]^. We learn here that the vasa vasorum of SV initiates from its feeding vessels in the adventitia, which then enter external and medial layers of the media, and form a complex spatial network of circular, longitudinal, and perpendicular oriented arterial and venous vessels of various orders, as well as capillaries (3.3 µm - 6.5 µm), where the latter can be present as far as the innermost parts of the media^[6,10]^. Apart from its well-known role in distributing and exchanging a variety of substances, the complex spatial arrangement of the vasa vasorum helps maintain vein elasticity when the vein is subjected to stretching or intraluminal pressure changes^[10]^. SEM studies also showed that the SV vasa vasorum can be structurally damaged by CON harvesting^[5].^ Additionally, TEM studies provided data about subcellular features of the SV wall, including its vasa vasorum^[4,12-14]^. These studies added to our better understanding of the anatomy of the vasa vasorum, and its response to harvesting procedures, therefore pointing to possible functional implications for graft performance. Indeed, clinical studies revealed the superiority of the long-term patency of NT-SV compared to CON-SV grafts^[15-17]^. It seems reasonable to claim that better preservation of the vasa vasorum in NT-SV grafts contributes to improved graft performance. Needless to say, the main role of vasa vasorum is to deliver blood with its plethora of components, including oxygen, to the wall of the host vessels^[18-20]^. Therefore, the details of structural and functional adaptations of the vasa vasorum in SV as coronary graft are particularly important.

## VASA VASORUM AND GREAT SAPHENOUS VEIN LUMEN

**Fig. 2:**
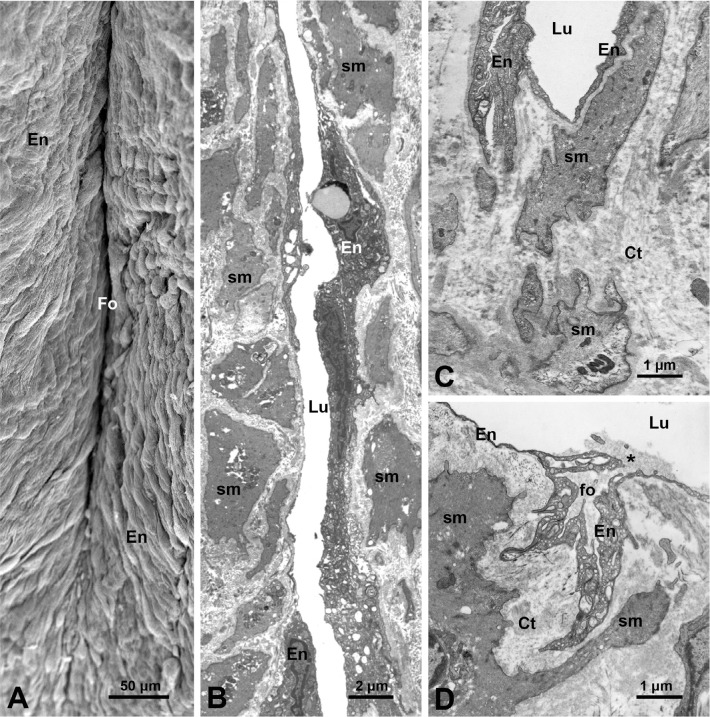
Scanning (SEM) and transmission (TEM) electron microscopy of human great saphenous vein harvested by the no-touch (A - C) and conventional methods (D). A) SEM image shows the luminal aspect of an inward intimal fold (Fo) between two segments of the inner media that is covered by the endothelium (En). B) TEM image shows an inside view of a fragment of a Fo with its lumen (Lu) and En; on either side of Lu, smooth muscle cells (sm) and connective tissue (Ct) of the media are present. C) TEM image shows a bottom region of a Fo; note the absence of the vasa vasorum vessels in this region. D) TEM Image shows the luminal aspects of conventionally harvested vein displaying a small branching fold (fo), protruding into the media. Note that the fo lumen is an extension of the vein Lu, as also is En. At the opening (~ 0.5 µm) of this fo note a clump (asterisk) of the blood components. It is acknowledged that A) image is modified from Vasilakis et al.[5], 2004; B), C), and D) are from A. Loesch unpublished study.

One of the findings that may be related to the vasa vasorum is the observation at LM and SEM levels of blood vessel-like profiles near SV lumen, suggesting that these could be the vasa vasorum terminating in the vein lumen^[3,21]^. But this assumption may or may not be correct. These blood vessel-like profiles might, in fact, be peripheral regions of SV folds (or subfolds), as can be seen later that were sectioned tangentially or in various plans. At LM level, the transversally sectioned SV (or NT-SV) grafts display several deep inward folds (~ 9 - 11) between the segments of the media, which bulge into lumen, and is covered by the intima^[4,5]^; these folds run along the longitudinal axis of the vein. Here Figure 1A shows well-defined luminal folds in NT-SV graft; while Figure 1B shows CON-SV graft, where luminal folds are less prominent due to distention during harvesting. It is not unusual for the peripheral regions of the media segments in NT-SV to be in tight contact with each other, sometimes giving a morphological impression of being a blood vessel (a vasa vessel) linked to the SV lumen^[3,21]^. These blood vessel-like profiles are positive for endothelial markers, including cluster of differentiation 31 (or CD31, a transmembrane highly glycosylated protein), cluster of differentiation 34 (or CD34, a transmembrane phosphoglycoprotein), and endothelial nitric oxide synthase. This indicates that they share the same antigenic characteristics as the intima of SV lumen^[12,21,22]^. Here Figures 1C to E demonstrate LCM images of intimal immunoreactivity for inducible nitric oxide synthase (iNOS). The blood vessel-like profiles described above, which are part of the intima of the fold, are iNOS-positive in both in NT-SV (Figure 2C) and CON-SV (Figure 2D). Note that there is no connection between the iNOS-positive intima and adventitial vasa vasorum (Figure 2E). It should be mentioned that our own TEM and SEM studies^[4,5]^ did not reveal any blood vessels or blood vessel-like profiles projecting into the intima of the SV lumen, including the peripheral and/or bottom regions of the luminal folds. The closest vasa vasorum vessels to the SV lumen were located at some 60 - 70 µm away^[5]^. Here Figures 2A to D show some ultrastructural features of folds in NT-SV grafts observed at both at SEM and TEM levels (Figures 2A and 2B, respectively), while Figure 2C presents a base region of a fold, and notably no blood vessels are present there. In addition to the deep main luminal folds, which have base parts facing the external layer of the media, the SV also displays much smaller fold structures. At TEM level, these appear as small branching folds protruding into the inner media, as can be seen in distended CON-SV grafts (Figure 2D). Again, no blood vessel profiles are observed in the vicinity of these small folds, indicating that they are not connected to the vasa vasorum system.

**Fig. 3:**
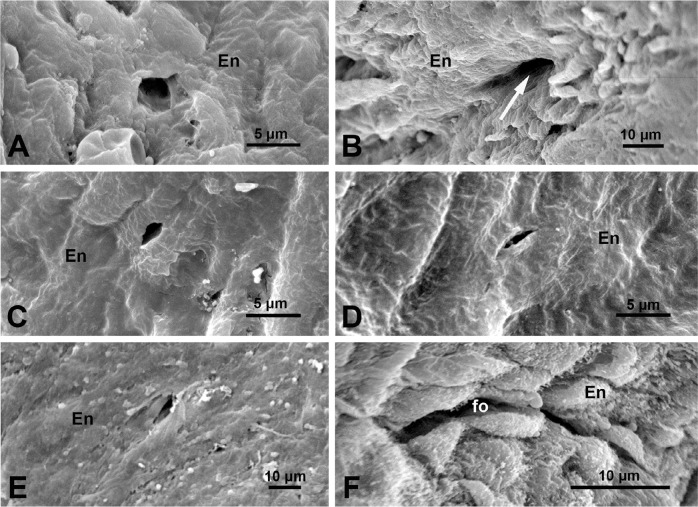
Scanning electron microscopy of luminal intima of a human great saphenous vein harvested as graft with the no-touch method. A) An intimal opening (~ 5 µm) is seen within the endothelium (En); the possibility was considered that the opening was a part of the vasa vasorum^[12]^. B) A blood vessel opening (> 10 µm), possibly a vasa vasorum or more likely a tributary branch that opens at the graft lumen. The arrow indicates a possible direction of blood flow on completion of coronary artery bypass grafting. C-E) Note examples of small (< 5 µm) intimal openings (‘channels’) of various shapes. F) An elongated opening perhaps to a small fold as illustrated in Figure 2D. It is acknowledged that A) image is from Dreifaldt et al.^[12]^, 2011; B) is modified from Loesch and Dashwood^[13]^, 2021; C), D), and E) are from A. Loesch unpublished study; and F) is modified from Vasilakis et al.^[5]^, 2004.

The adverse luminal and endothelial morphological changes induced by CON harvesting have previously been described at SEM and TEM levels^[2-5]^. Our recent SEM studies of the luminal aspect of SV graft preparations also revealed some new findings, sporadic appearance of small structures (Figure 3A), which were considered to be potential openings of the vasa vasorum in the intima of NT-SV grafts^[12]^. Further findings were more promising in this regard, as some of the structures displayed characteristics of vessel structures, such as the opening of a small vessel donating to the SV lumen^[13]^ (Figure 3B). It seems rather obvious that such vessel-like occurrence is part of a vascular system, but it remains difficult to determine whether this is an opening of a vasa microvessel or a small branch of a tributary that opens in SV lumen; the latter seems more plausible. This review presents more examples of small structures in the form of openings and/or channels of about 5 µm for the first time. These openings can be observed in the intima of NT-SV grafts (Figures 3C to E). Again, no evidence has been found that these openings are parts of the vasa vasorum system, even if the diameter of some falls within the range of vasa microvessels and/or capillaries (4.7 µm to 11.6 µm) previously found in human SV^[7]^. It must be stressed that no direct terminations (openings) of vasa vasorum to SV lumen were observed in studies of SV corrosion casts or veins injected with India ink^[6-8,11]^. Another form of intimal openings is present in the SV luminal intima. At the SEM level, these display rather narrow, elongated, and/or curved lumens of about 3 µm by 30 µm (Figure 3F). It is likely that such structures are the openings of the small folds (subfolds) in the inner media, as observed at TEM level (Figure 2D). The examination of Factor VIII staining in cross-sections of non-varicose cadaverous SVs, which were injected with India ink via the external iliac artery, revealed numerous Factor VIII-positive vasa vasorum in the deep layers of the media medium, including those closest to the hyperplastic intima^[8]^. A study of embalmed cadaverous SVs disclosed another important fact: the venae vasorum drain into the terminal segments of the largest tributaries of the SV lumen^[6]^. Thus, it can be assumed that the physical communication between the SV vasa vasorum and the SV lumen exists via the terminal segments of tributaries. This communication might play an important role after the completion of CABG as discussed in the next section.

## MYSTERY OF RETROGRADE BLOOD FLOW

At the time of completion of distal anastomosis during CABG, when checking for leakage before the proximal anastomoses are completed, the phenomenon of retrograde blood flow into an SV graft wall can be observed. This phenomenon is particularly pronounced in NT-SV grafts^[12,23]^. In fact, pressurized pulsatile bleeding can be seen from an incised blood vessel/s in the intact adventitia and/or perivascular tissue of NT-SV graft^[12]^. This intense bleeding is likely due to the volume of arterial blood entering the structurally preserved graft wall and its vasa vasorum system. Note that the abluminal site of SV carries the primary venous branch of vasa vasorum, which runs along the longitudinal axis of SV (for details of the structural arrangement of vasa vasorum, including its abluminal location in SV see the reference list^[9-11]^). Since the venules of the vasa vasorum empty into veins in the perivenous connective tissue^[24]^, the refilling with blood and/or bleeding from these veins when incised is visible^[12]^. Apart from the actual vessel opening to the SV lumen presented in Figure 3B, other small luminal openings (Figures 3A and C to E) are unlikely to be a part of vascular system, including that of the vasa vasorum, even though the potential contribution of such luminal openings to the retrograde blood flow into the graft has previously been suggested^[12]^. The most likely route for the aortic blood flow into the SV graft wall appears to be primarily via the terminal segments of tributaries to which the vasa vasorum, such as venae vasorum, donates^[6]^. It can be further speculated that the quantity of arterial blood entering the SV graft depends, in part, on the number and health of tributaries in the graft. In this case, the NT-SV grafts would have a clear advantage over CON-SV grafts due to being undamaged by harvesting^[2]^. It should be noted that the SV receives multiple tributaries along its course, with their number varying along the length of the vein^[24,25]^. This also implies that the health of a specific segment of the vein designated as the graft is crucial for the graft performance.

But the question remains unresolved regarding the properties of the blood circulation in the graft’s vasa vasorum system, as there is no direct link between the graft's vasa vessels and those of the host coronary artery directly after CABG. This raises the issue of aortic blood inflow and outflow in the newly implanted graft's vasa system. Could at least three phenomena be considered after completion of CABG? (1) Arterial blood in the graft lumen enters the graft wall via a tributary (or tributaries) located proximally to the direction of blood flow in the graft lumen; (2) blood exits the graft wall via a distally located tributary (or tributaries), returning to the graft lumen; and (3) blood pressure is increased in the vasa vasorum due to (a) ligated tributaries/branches (at ~ 0.5 cm from the vein wall in NT-SV grafts^[3]^), and (b) the lack of connectivity between of the graft’ vasa vasorum with the vasa system of the host coronary artery.

In general, different haemodynamic seem to apply to the vasa vasorum of the implanted SV graft compared to the vasa vasorum in the intact SV. One may ask question about the regulation of the blood flow in the vasa vasorum of SV graft and the properties of this flow. These attributes are particularly important in the early physiological stages of the graft following CABG, as they may help protect the graft wall from hypoxia. In the early phase of the graft after implantation, the roles of feeding and draining vessels associated with the SV vasa vasorum^[10,11]^ may be less relevant. It should be noted that no filling of the vasa vasorum with India ink was observed when India ink was injected into the lumen of SVs from cadavers^[6]^. This contrasts with the result of luminal India ink perfusion of SV segments *in vitro*, which showed a spread of India ink into the capillary network within the SV wall, including the pedicle of NT-SV but not CON-SV preparations^[26]^. The intramural vasa vasorum blood flow is complex, and there is no simple way of measuring it. However, if such comparison was possible, it would be interesting to compare NT-SV with CON-SV grafts in this respect. One of the methods that could be applied for such a comparison is the use of microspheres, as these were used to evaluate the effects of hypoxia on blood flow through arterial and venous vasa vasorum in dogs^[27]^. It can be stressed here that the dynamic of vasa vasorum is a complex phenomenon, sensitive to changes in tension of the host vascular wall. As a result, the dynamics of the vasa vasorum are linked with the quality of the vascular wall perfusion. In some vessels, this involves both vasa vasorum externa (entering the host vessel from its abluminal site) and vasa vasorum interna (entering the host vessel from its luminal surface)^[28]^.

## POST-CORONARY ARTERY BYPASS GRAFTING VASA VASORUM: PROLIFERATION

Following CABG, some complex processes may arise within the SV graft, leading to adverse outcomes such as formation of neointima and plaques and ultimately graft failure^[29-32]^. This subject is highly relevant to the patency of SV as a coronary graft^[33]^. Needless to say that the process of graft failure involves a variety of stimuli, with hypoxia being the most prominent. Neovascularization mechanisms are triggered, involving a number of pro-angiogenic growth factors like vascular endothelial growth factors, as well as inflammatory cells, cytokines, macrophages, and platelets^[30,34-36]^. It is thus clear that the issues of harvesting, vasa vasorum, hypoxia, and plaque formation are interlinked. This raises the question of why some SV grafts are more patent than the others, for example, when comparing the performance of NT *vs.* CON grafts^[15]^, and how the success or failure of the graft is related to the vasa vasorum system?

There is an important morphological study of explanted failed SV as coronary grafts, some up to 35 years after CABG^[37]^. The study reported a steady process of proliferation of vasa vasorum, initially from the adventitia to the outer media (7 - 14 months after CABG), and over time the vasa vasorum expanded to the entire atherosclerotic media and hyperplastic intima, finally expanding into plaques. Consequently, the authors conclude that vasa vasorum proliferation is a secondary reaction to the structural changes of the graft wall. However, the study does not specify how the SV grafts were harvested for CABG (up to 35 years before explantation), such as whether the grafts were stripped or distended during harvesting. However, the overall picture of the histological preparations of the explants suggests that the study was carried out on CON-harvested SV grafts. It is thus possible that the “massive” proliferation of vasa vasorum observed in explanted SV grafts^[37]^ resulted from the grafts’ response to compensate for the damage to native vasa vasorum inflicted by harvesting. Currently, we have no histological data on the status of proliferating (if any) vasa vasorum in explanted NT-SV grafts. Nevertheless, there are strong indications suggesting that the preservation of vasa vasorum in NT-SV grafts plays a protective role against hypoxia^[12,23]^. Such NT-SV grafts have also been shown to have preserved vasoconstriction and relaxation properties^[38]^, which may contribute to their superior patency rate compared to CON-SV grafts^[15,17,39-42]^.

## VASA VASORUM: AN INTIMATE RELATION

In the light of the abovementioned data^[37]^, the vasa vasorum proliferation begins at the abluminal (adventitial) site of the SV graft, which then spreads to the media, intima/neointima, and then to plaque. It is important mentioning here the original study of oxygenation profiles in the superficial femoral arteries of Yucatán miniature pigs, which demonstrated the initiating role of hypoxia in vasa vasorum proliferation^[43]^. Since then, numerous studies have clearly pointed to the adventitial site, including the adventitial blood vessels and vasa vasorum, as the primary sources of the vasa vasorum proliferation^[29,30,44,45]^. From the graft pathophysiological perspective, the phase of vasa vasorum proliferation from the media to the intima seems particularly noteworthy as it appears to be a critical contributor to the formation of neointima and plaque. A study of intimal microvessels in human coronary atherosclerosis revealed that these may leak plasma components into the arterial wall, thereby contributing to coronary atherosclerosis^[46]^. Indeed, microvascular leakiness, most likely due to a compromised endothelial barrier, and the dysfunction of microvessels play a role in initiating the complex processes that lead to atherosclerosis^[32]^. In comparison, damage to the intima, including the endothelial detachment and the exposure of subintimal matrix observed in CON-SV graft preparations^[3-5]^, makes these grafts particularly vulnerable to atherosclerosis.

One may question the role of small openings observed at SEM level in the luminal intima of NT-SV graft preparations (Figures 3A and C to E). It can be speculated that these openings serve as the “communicative channels” between the lumen and sub-endothelial matrix of the SV intima, enabling the exchange of circulating factors relevant to physiological or pathological conditions. In pathology, for instance, these "channels" could assist in the spread of newly formed microvessels. However, it cannot be ruled out that some of these openings are artefacts due to SEM procedures. If these intimal openings are genuine, they likely serve a functional purpose. It is well-known that vascular endothelial cells naturally renew when damaged or aged. This along with other morphological features of the luminal compartment, such as the presence of main folds and small folds protruding to the media, adds to the structural and morphological complexity of the SV intima. The SEM images presented in this review show that the intima is morphologically diverse. It can also be mentioned that the native SV, like other veins subjected to low transmural pressure, is plastic and can relatively easily change shape (vein deformability), allowing it to adapt to various physiological or pathophysiological conditions^[24]^. This deformability of the SV wall also may affect the intima and blood flow into the vein wall. Interestingly, the small folds projecting into inner media may help the vein stretch, which benefits CON harvesting. However, these structures may also play a role in varicose vein formation, contributing to the stretching and thinning of the vein wall media, and trapping blood in these small folds. In the other words, these small folds might represent “weak” sites in the media and SV wall.

To finish this section, it can be recalled that various studies have explored the possibility of the association between the intimal openings and the vasa vasorum, suggesting their potential involvement in the phenomenon of retrograde blood flow into the SV graft wall upon completion of CABG^[12,13]^. However, while this idea has been proposed, there is still no definitive evidence supporting the notion that these openings are direct extensions of the vasa vasorum, terminating in the SV lumen. Even if such extensions were present, it is unlikely that they would be sufficient to distribute the intense retrograde aortic blood flow observed in the graft wall during CABG^[12]^. As a matter of interest, over 50 years ago, an early observation on the use of the SV for arterial reconstruction stated: “The vasa vasorum in the arteries only penetrate to the medium, whereas the SV vasa vasorum are 5-8 times more numerous and penetrate through the entire wall thickness into the lumen”^[18]^. This statement, as we understand today, can be correct in relation to pathologically occluding SV as coronary graft^[37]^.

## CONCLUSION

Based on SEM, TEM, and LCM observations, no direct connection between the vasa vasorum and the lumen of the SV has been revealed. The intima of SV displays a variety of openings, ranging in size from about 5 µm to 20 µm. Among these, larger openings (of about 10 µm - 20 µm) are thought to be a part of blood vessels, such as branches of tributaries, rather than terminals of the vasa vasorum. The smaller openings, ranging from about 5 µm to 10 µm, appear to be non-vascular structures, potentially involved in subtle intima-subintima communication. In contrast to these openings, narrow, elongated intimal openings measuring about 3 µm by 30 µm are also present, which likely represent the entrances to small folds (subfolds) in the inner media of the vein. The issue of communication between the SV’s vasa vasorum and the vein lumen is particularly relevant to understanding the physiology of the SV as coronary graft, especially in the early stages following CABG.

## Data Availability

The author declares that the data supporting the findings of this study are available within the article.

## Artificial Intelligence Usage

The author declares that no artificial intelligence tool was used in the preparation of this article.
